# Engaging Pharmacists in Interprofessional Programs to Support Older Residents of HUD-Assisted Housing

**DOI:** 10.1093/geroni/igaa057.2746

**Published:** 2020-12-16

**Authors:** Patricia Slattum, Elvin Price

## Abstract

Medications are one of the pillar 4Ms of an age-friendly healthcare system. Ensuring that benefits of a medication regimen outweigh the risks and that medications are not contributing to meaningful outcomes such as functional or cognitive decline is a challenge for our healthcare system, particularly for older persons experiencing adverse social determinants of health, health disparities or uncoordinated care. More fully engaging pharmacists in the older person’s health care team is one strategy to improve clinical, humanistic and economic outcomes. This workshop reports findings on medication management capacity among older residents of five low-income apartment buildings in urban Richmond, VA, indicating that this population experiences challenges in managing medications as measured by MedMaIDE. The second presentation describes a pilot partnership between an independent community pharmacy and a local area agency on aging to provide medication coaching to residents of six affordable housing buildings in Seattle, WA, demonstrating feasibility of engaging pharmacists in the healthcare team and resulting in an average of five interventions per resident. The third presentation describes an interprofessional educational intervention based in two low-income apartment buildings for older persons in underserved West Baltimore, MD involving pharmacy students, to better prepare pharmacists to engage in interprofessional team care and meet the needs of a low income culturally diverse older adult population. Discussion will focus on barriers and opportunities to more fully engage pharmacists to support urban residents of low-income housing buildings to optimize medication outcomes and reduce medication-related harm.

